# Cloning, characterization and functional analysis of *NtMYB306a* gene reveals its role in wax alkane biosynthesis of tobacco trichomes and stress tolerance

**DOI:** 10.3389/fpls.2022.1005811

**Published:** 2022-10-06

**Authors:** Jing Yu, Bo Lei, Huina Zhao, Bing Wang, Kaleem U. Kakar, Yushuang Guo, Xiaolian Zhang, Mengao Jia, Hui Yang, Degang Zhao

**Affiliations:** ^1^Key Laboratory of Plant Resources Conservation and Germplasm Innovation in Mountainous Region (Ministry of Education), College of Life Sciences/Institute of Agro-bioengineering, Guizhou University, Guiyang, China; ^2^Guizhou Academy of Tobacco Science, Molecular Genetics Key Laboratory of China Tobacco, Guiyang, China; ^3^Department of Microbiology, Baluchistan University of Information Technology and Managemnet Sciences, Quetta, Pakistan; ^4^Plant Conservation Technology Center, Guizhou Key Laboratory of Agricultural Biotechnology, Guizhou Academy of Agricultural Sciences, Guiyang, China

**Keywords:** *NtMYB306a*, transcription factors, trichomes, *Nicotiana tabacum*, alkane biosynthesis

## Abstract

Trichomes are specialized hair-like organs found on epidermal cells of many terrestrial plants, which protect plant from excessive transpiration and numerous abiotic and biotic stresses. However, the genetic basis and underlying mechanisms are largely unknown in *Nicotiana tabacum* (common tobacco), an established model system for genetic engineering and plant breeding. In present study, we identified, cloned and characterized an unknown function transcription factor *NtMYB306a* from tobacco cultivar K326 trichomes. Results obtained from sequence phylogenetic tree analysis showed that *NtMYB306a*-encoded protein belonged to S1 subgroup of the plants’ R2R3-MYB transcription factors (TFs). Observation of the green fluorescent signals from NtMYB306a-GFP fusion protein construct exhibited that NtMYB306a was localized in nucleus. In yeast transactivation assays, the transformed yeast containing pGBKT7-NtMYB306a construct was able to grow on SD/-Trp-Ade+X-α-gal selection media, signifying that NtMYB306a exhibits transcriptional activation activity. Results from qRT-PCR, *in-situ* hybridization and GUS staining of transgenic tobacco plants revealed that *NtMYB306a* is primarily expressed in tobacco trichomes, especially tall glandular trichomes (TGTs) and short glandular trichomes (SGTs). RNA sequencing (RNA-seq) and qRT-PCR analysis of the *NtMYB306a*-overexpressing transgenic tobacco line revealed that *NtMYB306a* activated the expression of a set of key target genes which were associated with wax alkane biosynthesis. Gas Chromatography*–*Mass Spectrometry (GC-MS) exhibited that the total alkane contents and the contents of *n*-C28, *n*-C29, *n*-C31, and *ai*-C31 alkanes in leaf exudates of *NtMYB306a*-OE lines (OE-3, OE-13, and OE-20) were significantly greater when compared to WT. Besides, the promoter region of *NtMYB306a* contained numerous stress-responsive *cis*-acting elements, and their differential expression towards salicylic acid and cold stress treatments reflected their roles in signal transduction and cold-stress tolerance. Together, these results suggest that *NtMYB306a* is necessarily a positive regulator of alkane metabolism in tobacco trichomes that does not affect the number and morphology of tobacco trichomes, and that it can be used as a candidate gene for improving stress resistance and the quality of tobacco.

## Introduction

A well-integrated epidermis is critical for several important processes in plant development, shoot growth and plant defense (Javelle et al., 2011). The outmost epidermal layer of the plant leaf is composed of pavement cells, followed by specialized guard cells and trichomes, respectively (Bird and Gray, 2003). Trichomes are uni- or multi-cellular appendages resulting from divisions of epidermal cells, which primarily help in reducing evaporation by protecting the plant from wind and heat. Research has shown that trichomes found on leaves and stems accumulate and produce secondary metabolites in a species- and cultivar-specific manner, which provides forefront protection against herbivores, pathogens, and insects. Numerous studies have documented the important roles of trichomes in plant resistance to biotic and abiotic stresses, including drought and ultraviolet radiation (Wagner, 1991; Serna and Martin, 2006; McDowell et al., 2011; Glas et al., 2012). The trichomes of Tobacco (*Nicotiana tabacum* L.), an emerging model system for plant biology over the past several decades, are morphologically divided into three types: tall glandular trichome (TGT), short glandular trichome (SGT), and non-glandular trichome (NGT) (Wang et al., 2004; Huang et al., 2018; Wang et al., 2021b). In tobacco, many compounds such as cembranoids, sucrose esters, alkanes, and fatty acids are synthesized and secreted by trichomes, which are considered to be the main components of tobacco leaf exudates. In addition to inducing stress resistance in tobacco, they are also reported to be the precursors of important compounds produced in the process of leaf mellowing and are associated with the aroma of tobacco (Heemann et al., 1983; Johnson et al., 1985; Wagner, 1991; Sallets et al., 2014; Huang et al., 2018). Additionally, in flavology, alkanes produced from plant been shown to have a significant impact on flavor release and odor switch (Fu et al., 2021b). However, little is known about the key enzymes and associated genetic pathways involved in the biosynthesis of wax-alkanes in tobacco trichomes.

Plant epidermal waxes are complex mixtures of very-long-chain fatty acids (VLCFAs, with chain lengths of C20 to C34), alkanes, ketones, aldehydes, esters, and alcohols (Yeats and Rose, 2013; Jetter and Riederer, 2016). In *Arabidopsis thaliana*, the synthesis of these compounds occurs in endoplasmic reticulum, where four types of fatty acid elongase (FAE) complexes, including ketoacyl-CoA synthase (KCS), enoyl-CoA reductase (ECR), ketoacyl-CoA reductase (KCR), and hydroxyacyl-CoA dehydratase (HCD), catalyze the precursors of C16/C18 fatty acyls to form VLCFAs (Beaudoin et al., 2009; Javelle et al., 2011; Bernard and Joubès, 2013; Lee and Suh, 2015a; Xue et al., 2017). The first reaction catalyzed by KCS is the rate-limiting step of the FAE complex (Bird and Gray, 2003; Lee and Suh, 2015a). It is believed that KCS can specifically control the chain length of different substrates or products, while this specialty is not observed in the other three enzymes involved in chain extension (Hegebarth et al., 2016). VLCFAs serve as precursors for wax synthesis in plants, and the associated pathway split-up into two sub-pathways: the alkane synthesis pathway and alcohol synthesis pathway. The alkane synthesis pathway is also called the decarbonylation pathway, which mainly produces alkanes, aldehydes, ketones, and secondary alcohols (Samuels et al., 2008; Lee and Suh, 2015a; Pascal et al., 2019). So far, only a few genes involved in the wax-alkane synthesis pathway have been reported. Elongase 3-ketoacyl-CoA synthase encoded by *KCS1*, as one of the FAE complexes, is responsible for the extension of VLCFA chains (Todd et al., 1999). Two genes, *CER1* and *CER3*, encoding alkane synthetase and VLCFA reductase enzymes respectively, play a vital role in the biosynthesis of VLC-alkanes by controlling the reduction and decarbonylation of VLCFA-CoA to produce alkanes. Among these, the VLCFA reductase produces fatty aldehydes and catalyzes the conversion of very long-chain acyl-CoA to very long-chain alkanes, while alkane synthetase catalyzes the decarbonylation of fatty aldehydes to *n*-alkanes (Bourdenx et al., 2011; Bernard et al., 2012; Yeats and Rose, 2013; Xue et al., 2017; Pascal et al., 2019). Overexpression of *CER1* in *Poa pratensis* (Wang et al., 2021a) and cucumber (Wang et al., 2015) further confirmed the significant role of CER1 in alkane biosynthesis. In addition, *GDSL*, *ABCG*, and *HOTHEAD*, along with *CYP77A* and *CYP85A* belonging to the cytochrome P450 (CYP450) family, are all involved in the synthesis or transport of waxes and cutins to varying degrees (Panikashvili et al., 2011; Girard et al., 2012; Fabre et al., 2015; Lashbrooke et al., 2015; Nguyen et al., 2018; Shi et al., 2018; Tang et al., 2020). Research has shown that wax components in *Arabidopsis* trichomes are different from those of other epidermal cells, with higher concentrations of alkanes in trichomes. A study exhibited that *KCS* genes, such as *KCS1*, *KCS5*, and *KCS10*, were highly expressed in trichome cells, and may participate in the biosynthesis of wax components in trichomes (Hegebarth et al., 2016).

MYB transcription factors (TFs) are one of the largest TF families in plants and can function as gene activators. According to the number of MYB domains, the MYB TFs can be divided into four subfamilies including 1R-MYB, R2R3-MYB, 3R-MYB (R1R2R3-MYB), and 4R-MYB (Dubos et al., 2010; Liu et al., 2015). R2R3-MYB is the largest subfamily of MYB TFs, usually containing two MYB domains near the N-terminus and C-terminus. Based on sequence variation at the, R2R3-MYB members are further divided into 23 subgroups (Dubos et al., 2010), and members of the same subgroup show similar functions (Dubos et al., 2010; Jiang and Rao, 2020). R2R3-MYB TFs are involved in many plants physiological and biochemical activities, such as growth and development, primary and secondary metabolism, cell fate determination, and biotic and abiotic stress responses (Dubos et al., 2010; Liu et al., 2015; Jiang and Rao, 2020). It is known that members of the S1 and S9 subgroups of R2R3-MYB can regulate the synthesis of VLCFAs including waxes and cutins. For example, overexpression of MYB94, a member of the S1 subgroup, increased the total amount of waxes in *Arabidopsis* by activating the expression of genes (e.g., *KCS2* and *CER2*) related to the wax synthesis pathway (Lee and Suh, 2015b). MYB96, a transcriptional activator of wax biosynthesis in *Arabidopsis*, up-regulated the expression of *KCS1*, *KCS2*, *CER1*, and *CER3* (Seo et al., 2011). MdMYB30 bound to the *MdKCS1* promoter to activate its expression and regulate wax biosynthesis in apple (Zhang et al., 2019). In wheat, TaMYB31 has been reported to play a key role in drought stress tolerance by up-regulating the expression of wax biosynthesis genes (Zhao et al., 2018). The S9 subgroup members (i.e., MIXTA-MYB, AaMIXTA1 and MYB16) play an important role in controlling plant cell shape, epidermal permeability, and trichome development by regulating the expression of the structure and transport genes in the wax/cuticle biosynthesis pathway (Oshima and Mitsuda, 2013; Oshima et al., 2013; Shi et al., 2018; Jiang and Rao, 2020; Xu et al., 2021). Although, the members of the S1 subgroup have been well characterized in plants such as *Arabidopsis*, their roles in tobacco require further exploration.

In this study, we isolated and characterized a MYB TF, *NtMYB306a*, which was predominantly expressed in tobacco trichomes. Analyses of phylogenetic tree, protein sequence alignment, subcellular localization, and yeast transactivation assays indicated that NtMYB306a-encoded protein belongs to the S1 subgroup of MYB-R2R3 TFs and has transcriptional activation activity. The high expression of *NtMYB306a* in tobacco trichomes was further verified by quantitative real-time PCR (qRT-PCR), *in situ* hybridization, and GUS staining. In addition, the expression patterns of NtMYB306a under salicylic acid (SA), abscisic acid (ABA), and cold stress conditions were examined in tobacco. Finally, the target genes possibly activated by NtMYB306a were analyzed by RNA sequencing (RNA-seq) and qRT-PCR in tobacco plants overexpressing *NtMYB306a*. Gas chromatography-mass spectrometry (GC-MS) further detected the increase of alkane content in *NtMYB306a*-overexpression (*NtMYB306a*-OE) plants. Overall, our study provides a valuable reference and new insight for the regulatory network and candidate genes of the alkane biosynthesis pathway in tobacco trichomes.

## Materials and methods

### Plant materials and growth conditions

Tobacco (*N. tabacum* L.) cultivar K326 was used in this study. Tobacco plants were grown under 25 ± 2°C and 60% humidity conditions, with a photoperiod of 16/8 h light/dark.

### Isolation and characterization of *NtMYB306a*

In order to amplify *NtMYB306a* gene, PCR was performed using specific primers (**Supplementary Table S1**) and using tobacco trichomes that were obtained from frozen leaves according to a previous report (Zhang et al., 2018). The amino acid sequences of 126 R2R3-MYB proteins in *A. thaliana* were obtained from the TAIR database (http://www.arabidopsis.org/). A phylogenetic tree based on full-length amino acid sequences of R2R3-MYB proteins from tobacco and *A. thaliana* was constructed using MAGE v6.0. The neighbor-joining (NJ) method was performed with 1,000 bootstrap replications. Evolview v3.0 was used to further visualize the phylogenetic tree (Subramanian et al., 2019).

### Sample collection

To investigate the spatial and temporal expression patterns of *NtMYB306a*, we collected different tissues (roots, stems, flowers, leaves, leaves without trichomes, and trichomes) and leaves of different positions (the 1st, 4th, 7th, 11th, 15th, and 19th leaf, from the top to the bottom of the 5-month-old plant). All samples were frozen immediately in liquid nitrogen and stored at -80°C until RNA extraction, and three biological replicates were conducted for each sample.

### Hormone and abiotic stress treatments

Base leaves of 40-day-old tobacco plants were subjected to hormonal and cold stress treatments. The criteria of selection for the phytohormones and different treatment type was based on the *in-silico* detection of *cis*-acting elements in upstream regions of *NtMYB306a* gene. For hormone treatments, 10 μmol·L^-1^ ABA, 50 mg·L^-1^ SA, and 100 μmol·L^-1^ MeJA were sprayed on the leaves, respectively. For abiotic stress treatments, samples were subjected cold stress by transferring the plants to 4°C conditions. Samples were taken at 0, 6, 12, 24, 48, and 72 h post-treatment and immediately frozen in liquid nitrogen and stored at -80°C until further use. All treatments were performed with three biological replicates.

### RNA extraction and qRT-PCR analysis

Total RNA was extracted by using a plant RNA extraction kit (TransGen Biotech, Beijing, China) according to the manufacturer’s instructions. For the first-strand cDNA synthesis, a TransScript One-Step gDNA Removal and cDNA Synthesis SuperMix Kit (TransGen Biotech, Beijing, China) was used. qRT-PCR analysis was performed with TB Green Premix DimerEraser (TaKaRa, Dalian, China) on a ViiA7 Real-Time PCR Detection System (Applied Biosystems, USA), *β*-actin was used as the internal reference for normalization, and data were analyzed by using the 2^-ΔΔCT^ method (Livak and Schmittgen, 2001). The qRT-PCR program was as follows: 95°C for 30 s; followed by 40 cycles of 95°C for 5 s, 60°C for 30 s, and 72°C for 34 s. Then, a melting curve analysis was performed. Primers used for qRT-PCR are listed in **Supplementary Table S1**.

### Yeast transactivation assay

For the yeast transactivation assay, full-length open reading frame (ORF) of *NtMYB306a* was cloned and fused in the GAL4 DNA-binding domain of the pGBKT7 vector through *Nde I* and *Not I* restriction sites (primers are listed in **Supplementary Table S1**). The pGBKT7-NtMYB306a construct was transformed into yeast strain AH109 and grown on SD/-Trp, SD/-Trp-Ade, and SD/-Trp-Ade+X-α-gal selection media at 30°C for 3 d. Transactivation ability was determined by evaluating the growth of yeast cells on the selection medium.

### Subcellular localization

To determine the subcellular location of the NtMYB306a protein, the coding sequence (CDS) without the stop codon was ligated into the pBWA(V)HS-GLosgfp vector, which contains the CaMV 35S promoter and green fluorescent protein (GFP) gene, resulting in a fusion gene driven by the 35S promoter. Subsequently, this fusion vector and the control vector (NLS-mCherry vector that contains a red fluorescent protein) were electroporated into *A. tumefaciens* strain GV3101 and simultaneously infiltrated into the *N. benthamiana* leaves. After 48 h of infection, a Nikon C2+ confocal microscope (Nikon, Japan) was used to observe the green and red fluorescent signals. Primers used for plasmid construction are listed in **Supplementary Table S1**.

### *In situ* hybridization

Leaves of 60-day-old plants were used for *in situ* hybridization assay. Probes (5’-DIG-GUGUAAAAUUACCACCCUCAUCGACCGACAUG-DIG-3’) were designed and labeled with DIG-UTP. Tobacco leaves were fixed with FAA fixative, and the following procedures including embedding, section, hybridization, and development were performed as previously described (Choi et al., 2011). Leaf sections were then observed and photographed by a bright field microscopy.

### Molecular cloning of the *NtMYB306a* promoter and promoter-GUS fusion in transgenic tobacco plants

The 2,077 bp sequence upstream of the *NtMYB306a* translation start site was considered as the promoter region and amplified from the K326 genome, primers are listed in **Supplemental Table S1**. The *cis*-acting elements of the *NtMYB306a* promoter were analyzed using the PlantCARE website (http://bioinformatics.psb.ugent.be/webtools/plantcare/html/). The *NtMYB306a* promoter was constructed into the pCAMBIA1391Z vector and transformed into tobacco plants. Positive plants were identified by PCR, and GUS staining was conducted according to the method described by Jefferson et al. (1987). Tobacco tissues were immersed in the GUS staining buffer (0.5 mg/mL X-Gluc, 50 mmol/L sodium phosphate buffer [pH 7.0], 0.5 mol/L EDTA, 1% Triton X-100, and 40 mL/L methanol) at 37°C for 16-20 h, and then the material was dehydrated through an ethanol gradient and then photographed under a stereomicroscope.

### Tobacco transformation

Tobacco plants were transformed by the *Agrobacterium*-mediated method (Horsch et al., 1985), and the selection marker gene of the genetic transformation vector was hygromycin phosphotransferase (*HPT*). After three rounds of hygromycin (20 mg/L) selection on Murashige and Skoog (MS) medium, the regenerated buds were excised and rooted, and the T0 plants were self-crossed and harvested. The positive transgenic lines were screened on MS plates containing hygromycin (20 mg/L), and the expression level of *NtMYB306a* was detected by qRT-PCR. The T2 or T3 plants were used for molecular and metabolic analyses.

### RNA-seq and data analysis

Leaves were collected from 5-mouth-old plants including OE-20 T1 and WT plants, with three biological replicates per sample. Total RNA was extracted as described above. The following procedures were performed by Novogene Bioinformatics Technology Co., Ltd. (Beijing, China). Libraries were constructed and sequenced using an Illumina HiSeq 2500 platform. The RNA-seq raw data were transformed, filtered, and aligned to the *N. tabacum* cv.TN90 reference genome (https://www.ncbi.nlm.nih.gov/assembly/GCF_000715135.1/) (Sierro et al., 2014). FPKM of each gene was calculated based on the length of the gene and read counts mapped to this gene. Differential expression level, GO, and KEGG enrichment analyses of DEGs were performed on the Novomagic cloud platform (Novogene Bioinformatics Technology Co., Ltd., Beijing, China). DEGs were determined according to the following thresholds: |log2(fold-change)| > 1 and adjusted padj < 0.05.

### Analysis of alkanes in tobacco trichomes using GC-MS

According to Huang et al. (2018), leaves of 5-mouth-old tobacco plants were immersed in dichloromethane (CH_2_Cl_2_) solution three times for 2 s each time, which were combined and added with n-tetracosane (C24 alkane) as an internal reference. The extracted leaf exudate was vacuum-dried until most of the solvent was evaporated and the final volume reached 1 mL. The leaf exudate was passed through an organic filter membrane and analyzed by a GC-MS (7890B/5977B, Agilent, USA). Helium was used as the carrier gas, the flow rate was 1.0 mL/min; the temperature of the injection port was 250°C, and the injection volume was 1 µL; split injection was performed, and the split ratio was 10:1; the initial temperature was 50°C, hold for 1 min; from 50°C to 280°C at a rate of 5°C/min, hold for 1 min; then from 280°C to 300°C at a rate of 20°C/min, hold for 15 min; the total running time was 64 min. For the MS, the following parameters were used: MS detector; solvent delay: 3 min; ionization voltage: 70 eV; ion source temperature: 230°C; transmission line temperature: 280°C; scanning ion range: 35-450 amu; MS scanning mode: SCAN. The spectrum of each peak was analyzed by GC-MS using the mass spectrum database and the relative retention time of each peak, to determine the substance corresponding to each peak.

### Trichome count and SEM observation

Leaves of four randomly selected 5-month-old tobacco plants were collected for trichome count, and 5-8 mm^2^ pieces between the margin and middle rib were taken from leaves of the 10th to 12th positions, avoiding the midrib. Leaf pieces were fixed with 0.1 M phosphate buffer (pH 7.4) containing 2.5% glutaraldehyde for 24 h and 1% osmic acid for 2 h. After dehydration, replacement, drying, mounting, and gold spraying, a HITACHI Regulus 8100 SEM was used to observe the trichomes with an accelerating voltage of 3 kV, and the number of trichomes in the 1 mm^2^ area was calculated.

## Results

### Identification and characterization of *NtMYB306a* in tobacco

To identify the TFs that regulate important metabolites in tobacco trichomes, our research focused on the TFs that are predominantly expressed in trichomes. Therefore, the transcriptomic data (data not shown) of tobacco cultivar K326 trichomes was compared to those of leaves without trichomes. As a result, we identified 18 *MYB* genes that were predominantly expressed in tobacco trichomes, among which eight were annotated as *MYB306* TFs but with unknown function. It is reported that tobacco leaf exudates mainly include cembranoids, sucrose esters, alkanes, and fatty acids (Johnson et al., 1985; Sallets et al., 2014). Given that the trichomes are tiny specialized organs derived from epidermal cells, it was initially speculated that these *MYB306* genes, expressed predominantly in trichomes, may be related to the occurrence and development of trichomes, and the synthesis or transport of secondary metabolites.

**Figure 1:**
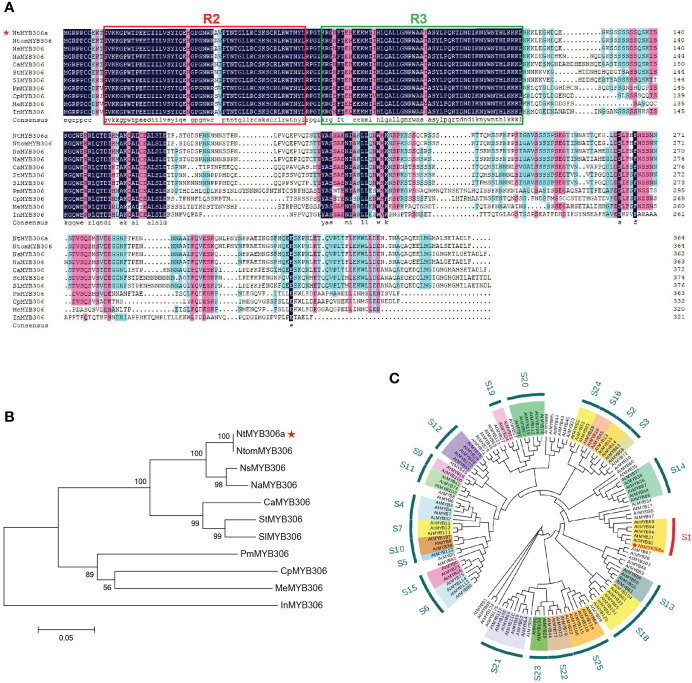
Multiple sequence alignment and phylogenetic analysis of the NtMYB306a protein and its homologs from various plant species. **(A)** Protein sequence alignment of MYB306 homologs. The R2 and R3 MYB DNA binding domains are indicated by red and green boxes, respectively. **(B)** Phylogram of NtMYB306a and related R2R3-MYBs from other plant species, including *Nicotiana tomentosiformis* (NtomMYB306: XP_009602669.1), *Nicotiana sylvestris* (NsMYB306: XP_009760429.1), *Nicotiana attenuata* (NaMYB306: XP_019239267.1), *Capsicum annuum* (CaMYB306: XP_016578359.1), *Solanum tuberosum* (StMYB306: XP_006354829.1), *Ipomoea nil* (InMYB306: XP_019198062.1), *Solanum lycopersicum* (SlMYB306: XP_004241580.1), *Prunus mume* (PmMYB306: XP_008239949.1), *Manihot esculenta* (MeMYB306: XP_021614785.1), and *Carica papaya* (CpMYB306: XP_021891841.1). *NtMYB306a* is highlighted with a red star. **(C)** Phylogenetic tree of NtMYB306a and *Arabidopsis thaliana* R2R3-MYBs. Individual subgroups are indicated with different colors. NtMYB306a, belonging to the S1 subgroup and being most closely related to AtMYB30, is highlighted with a red asterisk.

To identify the biological function of the *MYB306* gene, one of these genes from the cDNA of tobacco trichomes (accession number: *LOC107818868*) was cloned and named *NtMYB306a* (**Supplementary Figure S1**). Sequence analysis showed that the cDNA of *NtMYB306a* contained 1,095 nucleotides, encoding 364 amino acids (aa); the predicted molecular weight (MW) of the encoded protein was 40.46 kDa, and the theoretical isoelectric point (pI) was 6.29. Ten homologous genes of *NtMYB306a* from 10 other species were retrieved from the NCBI database using homology-based search. These homologous genes, together with *NtMYB306a*, were used to perform amino acid sequence alignment analysis and construct a phylogenetic tree (**Figures 1A, B**). Phylogenetic analysis of these 10 MYB306s revealed that *NtMYB306a* clustered with *NtomMYB306* (**Figure 1B**), and the similarity between the amino acid sequences of *NtMYB306a* and those of *NtomMYB306* and *NsMYB306* were 99.73% and 92.16%, respectively (**Figure 1A**). It is reported that *N. tabacum* has evolved from chromosome duplication after an interspecific hybridization of two diploid ancestors (i.e., *N. sylvestris* and *N. tomentosiformis*) (Leitch et al., 2008). The clustering pattern and close homology between *NtMYB306a* and *NtomMYB306* indicated that *NtMYB306a* may have originated from the diploid parent *N. tomentosiformis*. Moreover, *NtMYB306a* was found to share close homology to the *MYB306s* of other plants such as pepper, potato, and tomato, ascribing to the fact that these species belong to the *Solanaceae* family. The amino acid sequences of these MYB306s were highly conserved at the N-terminus that contained two DNA-binding domains, R2 and R3, with amino acids located at 13-63aa and 66-114aa, respectively (**Figure 1A**; **Supplementary Figure S2**). On the contrary, the C-terminal regions of the MYB306s were found to be more diverged. These results suggest that the *MYB306* genes are conserved during evolution, and NtMYB306a belongs to the R2R3-MYB subfamily. Members of the same subgroup of R2R3-MYB proteins often harbor similar biological functions (Dubos et al., 2010; Jiang and Rao, 2020). The phylogenetic tree constructed based on NtMYB306a and 126 *Arabidopsis* R2R3-MYBs showed that NtMYB306a belonged to the S1 subgroup of R2R3-MYB, and clustered with AtMYB30 (**Figure 1C**), with amino acid similarity of 45.53%. It has been shown that members of the S1 subgroup of plant R2R3-MYB proteins are regulators of wax and cutin biosynthesis (Seo et al., 2011; Lee and Suh, 2015b; Xue et al., 2017; Zhao et al., 2018; Zhang et al., 2019). Therefore, we speculated that *NtMYB306a*-encoded protein is likely to be involved in tobacco wax or cutin biosynthesis.

### Subcellular localization and transcriptional activation activity of NtMYB306a

**Figure 2:**
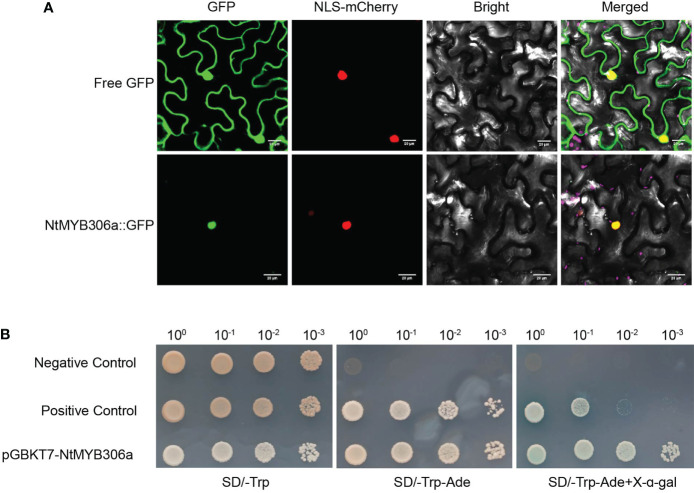
Subcellular localization and transcriptional activation of NtMYB306a. **(A)** NtMYB306a is located in the nucleus. GFP: green fluorescent protein signal; NLS-mCherry: red fluorescence indicates the nucleus-localized signal (NLS); Bright: bright field; Merged: GFP and NLS-mCherry overlay. Scale bars = 20 µm. **(B)** Transcription activation analysis of NtMYB306a protein in yeast. The pGBKT7 and pCL1 plasmids were used as negative and positive controls, respectively. 10^0^, 10^-1^, 10^-2^ and 10^-3^ indicate the yeast dilutions (1:1, 1:10, 1:100 and 1:1000).

Our above results revealed that *NtMYB306a* encodes a typical R2R3-MYB TF with transcriptional regulation activity (**Figure 1**), and suggested that NtMYB306a may function inside the nucleus. To investigate the subcellular localization of the NtMYB306a protein, a open reading frame (ORF) of the *NtMYB306a* without the terminator codon was fused to the GFP. The fusion protein construct was transiently expressed in *N. benthamiana* leaves and the GFP fluorescence of 35S: NtMYB306a-GFP was observed. These results demonstrated that the green fluorescence of NtMYB306a::GFP fusion protein completely coincided with the nuclear localized control vector (NLS-mCherry vector that contains a red fluorescent protein), indicating that NtMYB306a was localized in the nucleus (**Figure 2A**), which is also consistent with its role as a TF.

In addition to nuclear localization, transcriptional activation is another important feature of TFs. In the yeast transactivation assays (**Figure 2B**), the cells containing the pGBKT7-NtMYB306a plasmid was able to grow on SD/-Trp medium, signifying successful transformation. Furthermore, the yeast cells could grow on the SD/-Trp-Ade+X-α-gal selective medium and turned blue after 3 days. Simultaneously, the yeast cells transformed with the positive control pCL1 plasmid were able to grow on SD/-Trp-Ade medium, while those transformed with the negative control pGBKT7 were only able to grow on SD/-Trp medium. The above results indicate that NtMYB306a is a TF with transcriptional activation activity.

### *NtMYB306a* is predominantly expressed in tobacco trichomes

**Figure 3:**
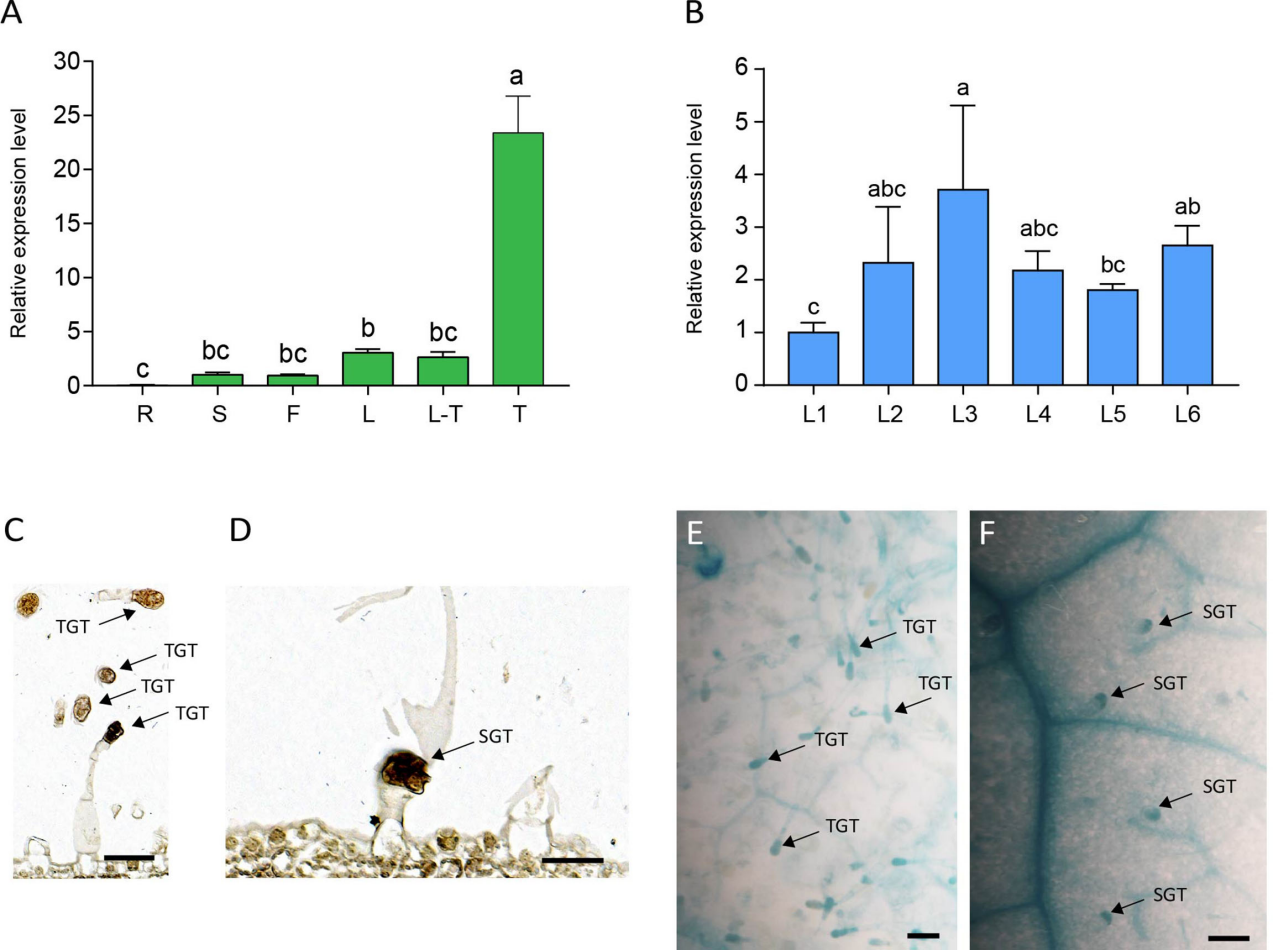
Spatial and temporal expression patterns of *NtMYB306a* in tobacco plants. **(A)** Relative expression levels of *NtMYB306a* in different tissues of tobacco plants. R, roots; S, stems; F, flowers; L, leaves; L-T, leaves without trichomes; T, trichomes. Error bars indicate the standard deviation (SD) of three biological replicates. Different letters above the bars indicate that those values are significantly different (P < 0.05) by one-way ANOVA. **(B)** Relative expression levels of *NtMYB306a* in leaves of different positions. L1-L6: the 1st, 4th, 7th, 11th, 15th, and 19th leaf position, respectively, from the top to the bottom of a plant. **(C, D)** mRNA *in situ* hybridization analysis of *NtMYB306a* in 60-day-old tobacco leaves. Strong signals were detected in tall glandular trichomes (TGTs) and short glandular trichomes (SGTs), which are indicated by black arrows. Scale bars = 50 µm. **(E, F)** GUS staining of pNtMYB306a:GUS transgenic tobacco plants. Black arrows indicate TGTs and SGTs, respectively. Scale bars = 100 µm.

To elucidate the expression pattern of *NtMYB306a*, various tobacco tissues were subjected to qRT-PCR analysis. As the data shown in **Figure 3A**, *NtMYB306a* was the highest expression level in trichomes, followed by leaves, flowers, and stems, respectively. Almost no expression was found in roots. The expression of *NtMYB306a* had no significant difference between leaves with or without trichomes. Additionally, we measured the relative expression levels of *NtMYB306a* in leaf tissues from different positions (i.e., L1-L6) in 5-months-old tobacco plants. The obtained data showed that the expression level of *NtMYB306a* in L3 leaves was higher than the leaves at other positions (**Figure 3B**). The expression level of *NtMYB306a* increasingly up-regulated from L1 to L2, peaked at L3, and started to decline at L4 and L5, respectively. At L6 position, the gene expression level was up-regulated to the level of L2 and L3. The *in-situ* hybridization analysis of *NtMYB306a* mRNA in 60-day-old tobacco leaves exhibited strong signal accumulation in tall glandular trichomes (TGTs) and short glandular trichomes (SGTs) (**Figures 3C, D**), which further indicated that *NtMYB306a* plays a role in tobacco glandular trichomes. In addition, strong hybridization signals were also observed in epidermis of some leaves (**Supplementary Figure S3**), which may be ascribed to the fact that trichomes are derived from epidermal cells. Next, we cloned the 2,077 bp long promoter sequence of *NtMYB306a* (**Supplementary Figure S4**) and built the pNtMYB306a:GUS construct to transform tobacco plants. In pNtMYB306a:GUS transgenic tobacco plants, GUS staining was clearly observed in TGTs and SGTs (**Figures 3E, F**). The above results indicated that *NtMYB306a* plays a key role in tobacco stems, leaves, and flowers, especially in trichomes.

### Promoter region of *NtMYB306a* harbour multiple stress-related *cis*-elements

**Figure 4:**
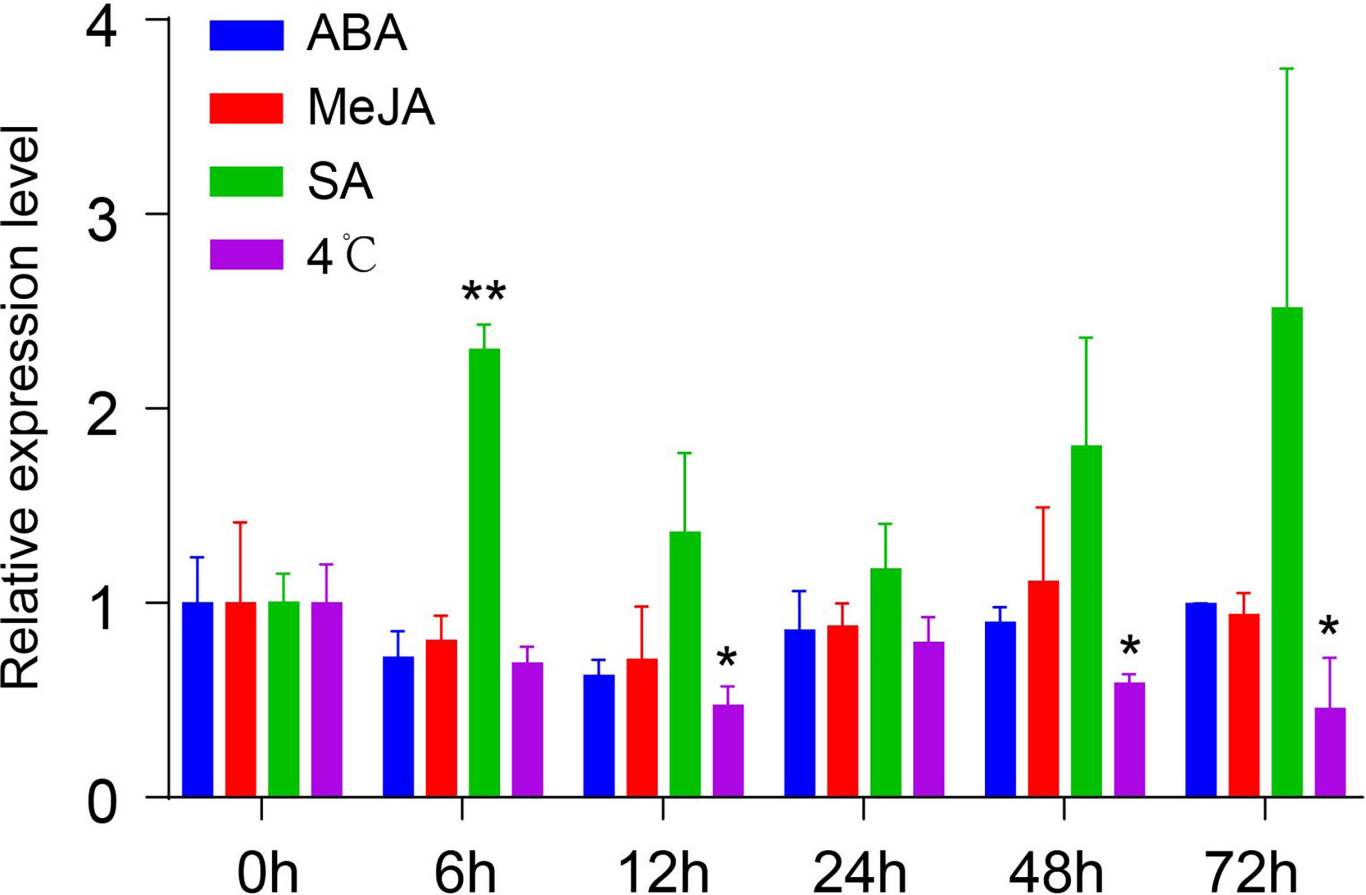
Relative expression levels of *NtMYB306a* in the leaves of tobacco plants under ABA, MeJA, SA, and 4°C treatments, respectively. Student’s t-test was used to analyze the difference between 0 h and other time points (*P < 0.05; **P < 0.01). Error bars indicate the SD of three biological replicates.

To understand the mechanism of transcriptional regulation and involvement of different regulatory components, the *cis*-acting elements of the *NtMYB306a* promoter were analyzed using the PlantCARE online tool. The *in-silico* analysis predicted numerous regulatory sequences present in the 5′ upstream regions of *NtMYB306a*, including ABA- (Abscisic acid), MeJA- (Methyle-jasmonate), SA- (Salicylic acid), light-, and cold-stress-responsive element (**Supplementary Table S2**). To compliment *in-silico* analysis of promoter regions, qRT-PCR experiment was designed to assess the expression of *NtMYB306a* in response to treatment with exogenous hormones (i.e., ABA, MeJA and SA) and cold stress (4°C) at different time points (0h, 6h, 12h, 24h, 48h and 72h). The results are shown in **Figure 4**. After ABA or MeJA treatment, no significant changes in the transcript abundance level of *NtMYB306a* were detected, while the *NtMYB306a* expression was significantly up-regulated after 6 h of SA treatment. At 12, 48, and 72 h of cold stress, the expression level of *NtMYB306a* was significantly down-regulated, showing negative co-regulation. The differential expression of *NtMYB306a* towards SA and cold stresses reflects complex regulatory mechanism during plant-environment interaction.

### Overexpression of *NtMYB306a* upregulates the transcription of wax/cutin synthesis-related genes

**Figure 5:**
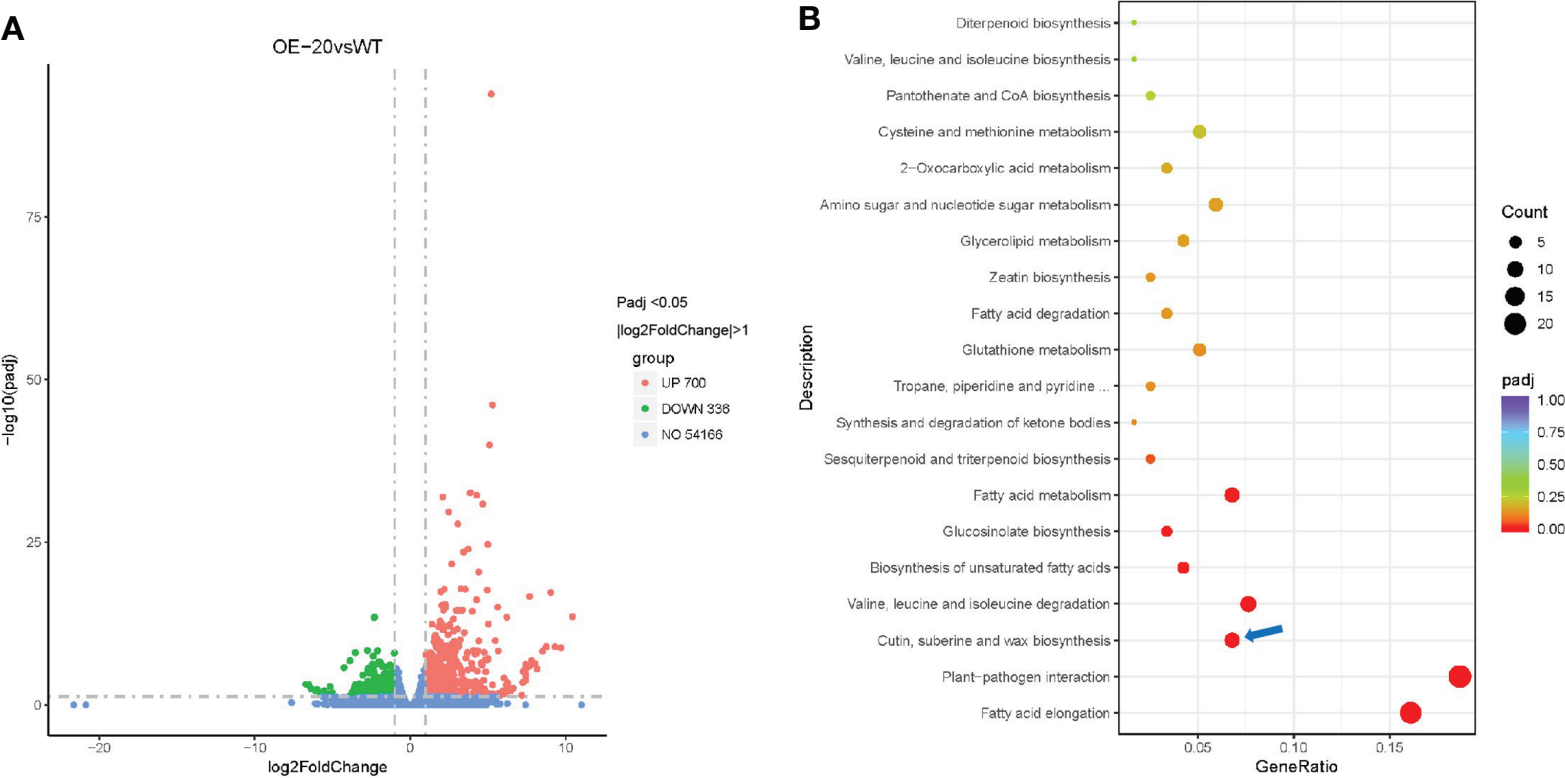
RNA-seq analysis of OE-20 and WT plants. **(A)** Volcano plot of DEGs between OE-20 and WT plants. Abundance of each gene was normalized as FPKM and DEGs were filtered for adjusted padj < 0.05 and |log2(fold-change)| > 1. DEGs shown in red color represent up-regulated genes (700), green showing down-regulated genes (336), and blue color indicates genes that were not differentially expressed (no-DEGs, 54,166). **(B)** KEGG enrichment analysis of up-regulated genes in OE-20 plants. The coloring of the padj-values represents the significance of the enrichment factor. Circles indicate the involved target genes, and the size is proportional to the gene number. The blue arrow indicates the cutin, suberine, and wax biosynthesis pathway.

To identify the key genes that may be regulated by NtMYB306a and explore the molecular mechanism, *NtMYB306a* was overexpressed in tobacco plants driven by the cauliflower mosaic virus (CMV) 35S promoter, and 11 *NtMYB306a*-OE transgenic lines (T0) were generated (**Supplementary Figures S5**, **S6**). To analyze the differential gene expression, RNA-seq analysis was performed on the leaves of OE-20 T1 transgenic and wild-type (WT) plants. The parameters for screening differently expressed genes (DEGs) were set to adjusted padj <0.05 and |log2(fold-change)|>1, which yielded a total of 1,036 DEGs. Compared with WT, 700 (67.57%) genes were up-regulated and 336 genes (32.43%) were down-regulated in OE-20 line (**Figure 5A**). Since NtMYB306a was predicted as TF with transcriptional activation activity, to have a further understanding on their function in biological system, the obtained 700 up-regulated DEGs were mapped to the Kyoto encyclopedia of genes and genomes (KEGG) database. As shown in **Figure 5B**, these DEGs were heavily enriched in biosynthesis of cutin, suberin, and wax. These results are consistent with our previous predictions based on the evolutionary tree, suggesting that NtMYB306a was related to wax and cutin biosynthesis. Genes that may be involved in wax and/or cutin biosynthesis are shown in **Table 1**. Among these, *GDSL* belonging to the lipase family, *ABCG32* belonging to the ABCG family, and P450 family members *CYP86A* and *CYP77A* are involved in cutin deposition or transport; *KCS* is involved in VLCFA biosynthesis; and *CER1* and *CER3* can convert VLCFAs into alkanes. In addition, genes involved in metabolic and/or biosynthetic processes (including glycerolipid, fatty acid, and unsaturated fatty acids), fatty acid degradation processes, and fatty acid elongation were also found to be enriched (**Figure 5B**). Valine and isoleucine are the precursors for generating alkanes (Grice et al., 2008), and genes related to the biosynthesis and degradation of valine and isoleucine were also enriched by KEGG analysis. In higher plants, alkanes are the decarboxylation products of fatty acids (Grice et al., 2008), indicating that NtMYB306a is involved in wax accumulation by regulating the transcription of lipid-related genes. In addition to genes related to lipid metabolism, plant-pathogen interaction pathways were also identified in KEGG enrichment analysis (**Figure 5B**), suggesting that NtMYB306a is also involved in plant defense.

**Table 1:** Genes involved in wax and/or cutin biosynthesis in NtMYB306a-OE (OE-20) plants.

Gene ID	Encoding proteins	Gene symbol	FPKM (OE-20)	FPKM (WT)	log2FoldChange
LOC107795401	GDSL esterase/lipase EXL3-like	GDSL	500.86	246.41	1.03
LOC107783455	GDSL esterase/lipase EXL3-like	GDSL	792.02	83.36	3.25
LOC107783245	GDSL esterase/lipase At2g04570-like	GDSL	2719.53	1041.40	1.39
LOC107786667	GDSL esterase/lipase At2g04570-like	GDSL	1250.90	399.98	1.65
LOC107810512	GDSL esterase/lipase At2g04570-like	GDSL	1105.73	155.50	2.83
LOC107832819	GDSL esterase/lipase At1g28610-like	GDSL	114.33	41.73	1.46
LOC107789302	GDSL esterase/lipase At1g28610-like	GDSL	91.61	4.67	4.30
LOC107829515	GDSL esterase/lipase APG-like	GDSL	2876.54	1015.95	1.50
LOC107769403	GDSL esterase/lipase APG-like	GDSL	1865.14	449.67	2.05
LOC107770381	GDSL esterase/lipase 2-like	GDSL	54.80	12.42	2.13
LOC107826741	GDSL esterase/lipase 6-like	GDSL	120.82	0.69	7.49
LOC107766261	GDSL esterase/lipase 6-like	GDSL	261.66	0.00	10.48
LOC107785441	GDSL esterase/lipase At4g16230-like	GDSL	17.32	0.64	4.73
**LOC107805226**	**GDSL esterase/lipase At4g16230-like%2C transcript variant X2**	**GDSL**	**570.13**	**0.72**	**9.71**
LOC107761143	cytochrome P450 77A1-like	CYP77A1	519.83	249.48	1.06
LOC107767679	cytochrome P450 71A1-like	CYP77A1	31.20	0.00	7.42
**LOC107764015**	**cytochrome P450 77A2**	**CYP77A2**	**388.61**	**20.88**	**4.22**
**LOC107788811**	**cytochrome P450 86A8-like**	**CYP86A8**	**149.00**	**2.94**	**5.65**
LOC107827543	cytochrome P450 86A22-like	CYP86A22	624.08	19.22	5.02
LOC107817982	3-ketoacyl-CoA synthase 1	KCS1	437.76	119.37	1.87
**LOC107794424**	**3-ketoacyl-CoA synthase 1-like**	**KCS1**	**480.42**	**45.92**	**3.39**
LOC107826268	3-ketoacyl-CoA synthase 3-like	KCS3	285.45	110.54	1.37
**LOC107831519**	**3-ketoacyl-CoA synthase 3-like**	**KCS3**	**591.07**	**61.38**	**3.27**
LOC107766412	3-ketoacyl-CoA synthase 5-like	KCS5	1111.99	313.50	1.83
LOC107810871	3-ketoacyl-CoA synthase 5-like	KCS5	4966.43	1335.33	1.90
LOC107784643	3-ketoacyl-CoA synthase 6-like	KCS6	473.70	249.19	0.93
LOC107814680	3-ketoacyl-CoA synthase 10-like	KCS10	1506.93	594.45	1.34
LOC107811181	3-ketoacyl-CoA synthase 10-like	KCS10	1578.24	442.33	1.84
LOC107820032	3-ketoacyl-CoA synthase 11-like	KCS11	137.36	43.70	1.66
LOC107785233	3-ketoacyl-CoA synthase 11-like	KCS11	84.00	15.53	2.42
**LOC107806171**	**3-ketoacyl-CoA synthase 11-like**	**KCS11**	**514.90**	**15.63**	**5.05**
LOC107802970	3-ketoacyl-CoA synthase 20-like	KCS20	843.61	338.02	1.32
LOC107780360	3-ketoacyl-CoA synthase 20-like	KCS20	229.97	51.82	2.15
**LOC107809103**	**3-ketoacyl-CoA synthase 20-like**	**KCS20**	**1275.27**	**198.51**	**2.69**
**LOC107793182**	**protein ECERIFERUM 1-like**	**CER1**	**626.03**	**252.92**	**1.31**
LOC107778616	protein ECERIFERUM 1-like	CER1	1202.65	449.78	1.42
LOC107772305	protein ECERIFERUM 1	CER1	472.44	161.77	1.55
LOC107829391	protein ECERIFERUM 3-like	CER3	1050.93	406.62	1.37
LOC107777290	protein ECERIFERUM 3-like	CER3	854.22	338.80	1.33
**LOC107792392**	**protein ECERIFERUM 3-like%2C transcript variant X1**	**CER3**	**1542.32**	**497.05**	**1.63**
LOC107787320	protein HOTHEAD-like%2C transcript variant X1	HOTHEAD	565.67	189.28	1.58
LOC107814122	protein HOTHEAD-like%2C transcript variant X1	HOTHEAD	143.89	44.81	1.67
LOC107767080	protein HOTHEAD-like	HOTHEAD	322.28	55.78	2.52
LOC107777350	protein HOTHEAD-like	HOTHEAD	1251.27	211.20	2.57
LOC107832333	ABC transporter G family member 15-like	ABCG15	1888.61	744.87	1.34
LOC107828913	ABC transporter G family member 15-like	ABCG15	4284.28	1436.33	1.58
LOC107802663	ABC transporter G family member 32-like	ABCG32	715.47	207.54	1.79
**LOC107767674**	**ABC transporter G family member 32-like%2C transcript variant X3**	**ABCG32**	**901.59**	**237.58**	**1.93**
LOC107831523	ABC transporter G family member 35-like	ABCG35	1184.97	345.26	1.78

Genes in bold font indicate the 10 genes used for qRT-PCR analysis.

**Figure 6:**
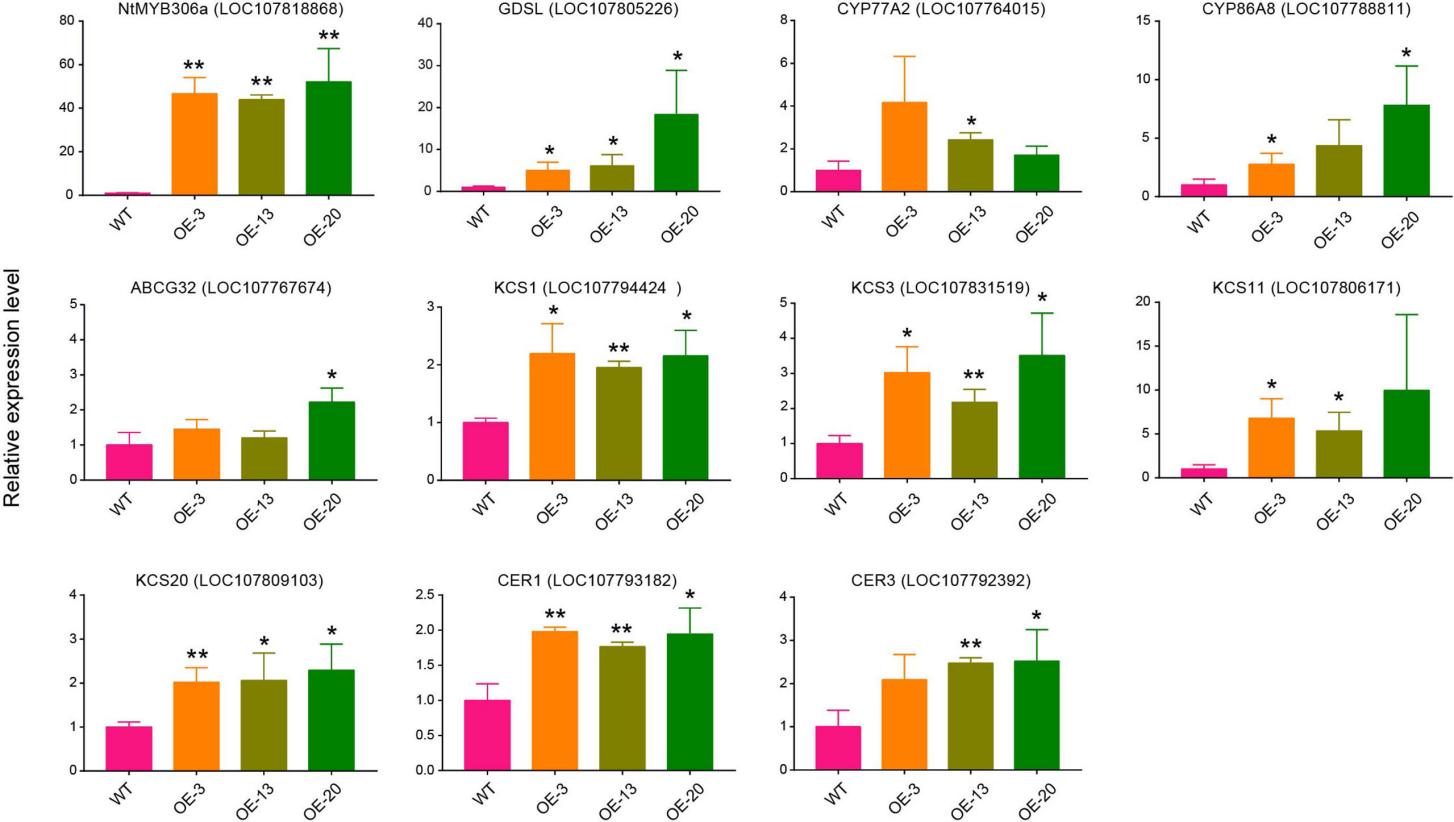
Relative expression levels of genes involved in the wax/cutin biosynthesis pathway in *NtMYB306a*-OE and WT plants. Ten putative target wax/cutin metabolism-related genes, including *GDSL*, *CYP77A2*, *CYP86A8*, *ABCG32*, *KCS1*, *KCS3*, *KCS11*, *KCS20*, *CER1*, and *CER3*, were selected. Error bars indicate the SD of three biological replicates. Student’s t-test: *P < 0.05; **P < 0.01.

To further confirm the possible target genes regulated by NtMYB306a, we performed qRT-PCR assay to investigate the expression patterns of ten putative target wax/cutin metabolism-related genes in the T2 plants of OE-3, OE-13, and OE-20 lines. The chosen target genes included *GDSL*, *CYP77A2*, *CYP86A8*, *ABCG32*, *KCS1*, *KCS3*, *KCS11*, *KCS20*, *CER1*, and *CER3*. As expected, their expression was induced in these transgenic lines (**Figure 6**), which was consistent with the data obtained from RNA-seq (**Table 1**). Nearly all lines had small increases in expression of target genes relative to wild-type. Comparison between over-expression lines, OE-3, OE-13 and OE-20, eight genes were significantly expressed in OE-20 and OE-13, while seven genes were expressed in OE-3 as compared to WT lines. The expression of *NtMYB306a* showed 40 - 60-fold increase in all three homozygous lines compared to WT. Among ten target genes, the highest level of expression was noted for *GDSL*, which was ~20 fold increased in OE-20, followed by *CYP86A8* (~7fold) and *KCS3* (~3fold) respectively. Other genes, including *CER1, CER3*, *KCS1*, *KCS11*, *KCS20, CYP77A2, ABCG32* showed lower yet significant level of expression in over-expression lines relative segregating wild-type plants. In addition, the expression level of these ten genes was examined in *NtMYB306a*-OE tobacco plants under the control of tobacco glandular trichome-specific promoter pCBTS (Ennajdaoui et al., 2010). In the pCBTS : *NtMYB306a*-T1 plants, the expression of the target set of genes showed similar significantly up-regulation trends (**Supplementary Figure S7**). These results suggest that NtMYB306a may be an activator of a set of genes related to wax and cutin biosynthesis in tobacco trichomes.

### Overexpression of *NtMYB306a* enhances alkane content in leaf exudates

**Figure 7:**
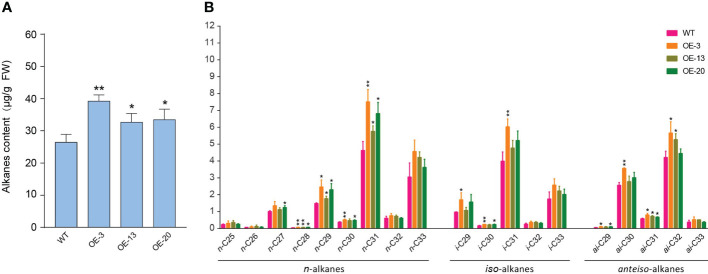
Alkane contents in *NtMYB306a*-OE and WT leaf exudates. **(A)** Total alkane content. **(B)** The content of *n*-alkanes, *iso*-alkanes, and *anteiso*-alkanes, respectively. Error bars indicate the SD of three biological replicates. Student’s t-test: *P < 0.05; **P < 0.01.

In *Arabidopsis*, the composition of surface wax on trichomes is different from other epidermal cells, and alkanes are the central components of trichome waxes (Hegebarth et al., 2016). In this study, NtMYB306a belonging to the S1 subgroup of R2R3-MYB was predominantly expressed in trichomes, indicating that NtMYB306a may be involved in the synthesis or secretion of alkanes in tobacco trichomes. Components of tobacco leaf exudates were surveyed by Gas Chromatography*–*Mass Spectrometry (GC-MS) that exhibited that the total alkane contents in leaf exudates of *NtMYB306a*-OE lines (OE-3, OE-13, and OE-20) were significantly greater than those of the WT (**Figure 7A**), and the contents of *n*-C28, *n*-C29, *n*-C31, and *ai*-C31 alkanes were significantly higher than those in WT (**Figure 7B**). In WT plants, *n*-C31 and *n*-C33 alkanes accounted for the majority of *n*-alkanes ranging between *n*-C25 and *n*-C33, and there was a small amount of *n*-C25 and *n*-C26, alkanes (**Figure 7B**). The contents of *i*-C31 and *i*-C33 contents among *iso*-alkanes and the *ai*-C30 and *ai*-C32 contents in *anteiso*-alkanes was relatively high in all samples. A similar result was reported by Grice et al. (2008), where the *n*-alkanes were found to be predominant in WT tobacco leaf exudates among the three homologs (i.e., *n*-alkanes, *iso*-alkanes, and *anteiso*-alkanes). Moreover, the trend for alkane contents presented in tobacco trichome samples is similar to that reported in *Arabidopsis* trichomes, where the *n*-C29 and *n*-C31 homologs were predominant in WT *Arabidopsis* samples (Hegebarth et al., 2016). These results further suggest that NtMYB306a is a positive regulator of alkane metabolism in tobacco trichomes.

### Overexpression of *NtMYB306a* does not influence tobacco trichome density

**Figure 8:**
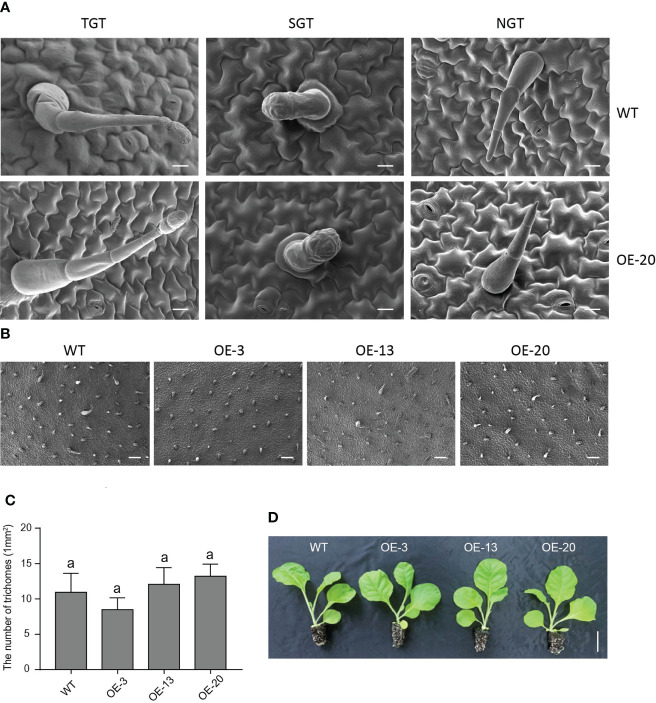
Phenotypes of *NtMYB306a*-OE and WT leaves. **(A)** The morphologies of tall glandular trichome (TGT), short glandular trichome (SGT), and non-glandular trichome (NGT) in OE-20 and WT plants. Scale bars = 20 µm. **(B)** Trichome abundance on WT, OE-3, OE-13, and OE-20 leaves, respectively. Scale bars = 20 µm. **(C)** The number of trichomes on WT, OE-3, OE-13, and OE-20 leaves. Error bars indicate the SD of four biological replicates. Different letters above the bars indicate that those values are significantly different (P < 0.05) by one-way ANOVA. **(D)** Phenotype observation of WT and three *NtMYB306a*-OE transgenic lines (OE-3, OE-13, and OE-20). Scale bars = 5 cm.

Trichomes are specialized organs of epidermal cells, and wax and cuticle biosynthesis is reportedly associated with the differentiation of epidermal cells, and the initiation and development of trichomes (Shi et al., 2018). Our scanning electron microscopy (SEM) images showed that there were no obvious differences in leaf surface structures between the TGTs, SGTs, NGTs, and pavement cells of the overexpression *NtMYB306a*-OE line (OE-20) and WT plants (**Figure 8A**). We observed that the numbers of leaf trichomes was not significantly different between *NtMYB306a*-OE lines (OE-3, OE-13, and OE-20) and WT (**Figures 8B, C**). Additionally, 2-month-old *NtMYB306a*-OE lines showed no significant difference in macroscopic phenotype compared with WT plants (**Figure 8D**). The above results indicate that overexpression of *NtMYB306a* in tobacco fails to alter the tobacco trichome density, and thus had no significant effect on the morphology of trichomes.

## Discussion

### *NtMYB306a* responds to SA induction and is predominantly expressed in tobacco trichomes

Trichomes are specialized tissues located on the epidermis of many plant species, that protect plant from excessive transpiration, and participate in resistance mechanisms to numerous abiotic stresses including high temperature, radiation and UV light, and biotic stresses such as pathogens and herbivore attack (Wagner, 1991; Serna and Martin, 2006; Glas et al., 2012). In tobacco, glandular trichomes act as sites for the biosynthesis and secretion of cembranoids and several secondary metabolites (Johnson et al., 1985; Wang et al., 2004; Ennajdaoui et al., 2010), hence they serve as important tool of genetic engineering for quality improvement of varieties (Glas et al., 2012; Tissier, 2012; Wu et al., 2020). However, limited studies to date have investigated the development of tobacco trichomes, and the detailed mechanism has not been fully understood. The present study was conducted to identify regulatory factors that may play a role in the metabolism of leaf trichomes in tobacco. Previous studies have shown that some TFs, which are predominantly expressed in plant glandular trichomes, are involved in the secondary metabolite synthesis of glandular trichome and trichome development. For example, artemisinin of *Artemisia annua* and menthol of spearmint are specifically synthesized and secreted in glandular trichomes. Among different genes discovered, *AaGSW1* (Chen et al., 2017), *AaWRKY9* (Fu et al., 2021a), *AaORA* (Lu et al., 2013), and *AaMYB15* (Wu et al., 2021) of *A. annua* regulate the artemisinin synthesis pathway, *AaTAR2* (Zhou et al., 2020) is involved in the regulation of trichome development, while, *MsMYB* is involved in the production of monoterpenes in spearmint (Reddy et al., 2017). In our current study, we isolated, characterized, and subsequently cloned the *NtMYB306a* (**Supplementary Figure S1**) gene from tobacco by comparing the transcriptomic data between leaves with and without trichomes. NtMYB306a was localized in the nucleus and showed transcriptional activation activity. Scores obtained from protein sequence alignment and phylogenetic tree analysis showed that NtMYB306a belonged to the S1 subgroup of R2R3-MYB TFs, which are involved in wax and cutin biosynthesis of plants (Seo et al., 2011; Lee and Suh, 2015b; Zhang et al., 2019; Wen et al., 2021). We further confirmed the predominant expression of *NtMYB306a* in tobacco trichomes, especially glandular trichomes, by employing qRT-PCR, *in situ* hybridization, and GUS staining, and speculated that *NtMYB306a* was involved in the biosynthesis or transport of wax and cutin in tobacco trichomes. In addition, it has been reported that the S1 subgroup members, such as AtMYB30, regulate the transduction of cell death-related lipid signals by enhancing the synthesis of VLCFAs or their derivatives (Raffaele and Rivas, 2013). Moreover, AtMYB30 plays an important role in plant defense and stress response (Marino et al., 2013; Raffaele and Rivas, 2013; Liao et al., 2017; Mabuchi et al., 2018), while SA is an important signaling molecule in plant defense (Ding and Ding, 2020). We found that *NtMYB306a* expression was induced by SA, and the RNA-seq data strongly supported the results, i.e., several stress-related DEGs were enriched in *NtMYB306a*-OE transgenic lines. These findings suggest that *NtMYB306a* can enhance VLCFA synthesis and participate in SA signal transduction.

### Up-regulation of wax/cutin-related genes increases the alkane content in *NtMYB306a*-OE lines

Through genome-wide transcriptome analysis, we revealed that many protein-coding genes involved in wax biosynthesis were regulated by the NtMYB306a. Considering that NtMYB306a showed transcriptional activation activity, we found that the expression levels of wax and cutin synthesis-related genes were up-regulated in *NtMYB306a*-OE lines. Among these, *KCS1*, *KCS3*, *KCS5*, *KCS10*, *KCS11*, and *KCS20* may be related to the elongation of VLCFAs (Todd et al., 1999; Lee et al., 2009; Bernard and Joubès, 2013; Xiong et al., 2020; Batsale et al., 2021), while CER3 and CER1 convert VLCFAs into alkanes (Bourdenx et al., 2011; Bernard et al., 2012; Pascal et al., 2019). In addition, CYP77A and CYP86A of the CYP450 family, which are involved in the biosynthesis of cutin monomers (Li-Beisson et al., 2009); and GDSL lipase (Girard et al., 2012; Tang et al., 2020) and ABCG protein (Panikashvili et al., 2011; Nguyen et al., 2018; Philippe et al., 2022), which are related to wax and cutin synthesis, transport, and secretion, were also up-regulated in *NtMYB306a*-OE plants. However, the functions of most of the above genes have not been characterized due to the lack of research on the wax and cutin synthesis pathways in tobacco. Therefore, we also performed an RNAi experiment on *NtMYB306a*, but we did not obtain *NtMYB306a*-RNAi lines with silenced *NtMYB306a* (data not shown), which might be due to the fact that cultivated tobacco is allotetraploid and has multiple structurally similar *MYB306* homologs. The predominant wax components of trichomes are mainly alkanes in *Arabidopsis* (Hegebarth et al., 2016); therefore, we next focused on detecting the alkane content in tobacco leaf exudates and observed an increase in the alkane content in *NtMYB306a*-OE plants. Combining with the results of RNA-seq and qRT-PCR, we speculate that NtMYB306a can activate the expression of several genes of wax and cutin biosynthesis pathways. These genes include those involved in VLC-alkane synthesis, thereby increasing the content of alkanes.

### NtMYB306a positively regulates genes in trichomes to synthesize and transport trichome-specific wax alkane components

We detected alkanes in tobacco leaf exudates, but whether these substances were specifically synthesized in trichomes or secreted by glandular trichomes after being synthesized by epidermal cells is unclear and requires detailed validation with further experiments. It is challenging to isolate the required amount of trichome metabolites from tobacco leaves, which limited accurate examination of trichome-specific wax components. However, results of some studies support the synthesis of specific wax alkanes in plant trichomes. For example, in *Arabidopsis*, all FAE complexes share the same KCR, HCD, and ECR compounds but different KCSs, which determine the specificity of substrates and product chain lengths of the entire complex (Hegebarth et al., 2017). Some cell-specific *KCS* genes, which are only expressed in trichomes or guard cells but not in the pavement cells of the epidermis (Samuels et al., 2008), may be involved in different pathways of trichomes (Hegebarth et al., 2017). For example, *KCS1*, *KCS5*/*CER60*, *KCS8*, and *KCS10* were mainly expressed during trichome development, which may be involved in the formation of trichome waxes (Hegebarth et al., 2016). Furthermore, *Arabidopsis* trichomes have higher concentrations of alkanes, which is consistent with the relatively high expression of two enzyme genes (*CER3* and *CER1*) involved in the alkane synthesis pathway in trichomes (Hegebarth et al., 2016). In our study, some genes were up-regulated after overexpression of *NtMYB306a*, such as *KCS1* (*LOC107794424*), *KCS3* (*LOC107831519*), *KCS20* (*LOC107809103*), *CER1* (*LOC107793182*), *CER3* (*LOC1077492392*), and *GDSL* (*LOC1078052767*). The expression levels of these genes were much higher in trichomes than in other epidermal cells (**Supplementary Figure S8**), further supporting that NtMYB306a positively regulates these genes in trichomes to synthesize trichome-specific wax alkane components. Collectively, these genes that are predominantly expressed in tobacco trichomes have not been characterized and are candidate genes related to wax alkane biosynthesis and transport in tobacco trichomes.

### Overexpression of *NtMYB306a* does not influence the number and morphology of tobacco trichomes

Some studies have shown that the R2R3-MYB TFs can affect epidermis development by activating the expression of genes related to wax and cutin biosynthesis (Shi et al., 2018), thus regulating the occurrence and development of trichomes. Most of these genes are members of the S9 subgroup, for example, in *Arabidopsis*, a MIXTA-like TF MYB106 regulates trichome branching by inducing cutin and wax biosynthesis (Oshima et al., 2013); in *A. annua*, AaMIXTA1 promotes GST initiation by regulating cuticle biosynthesis (Shi et al., 2018). These genes are closely related to the initiation and development of trichomes, which may be because the plant leaf epidermis is a mixture composed of cutins and waxes, and trichomes protrude from the epidermal cell layer (Bird and Gray, 2003). Some correlational studies have demonstrated the relationship among the S1 subgroup members of MYB, trichome development and wax synthesis. For instance, SlMYB31 interacted with the HD-Zip IV TF Woolly to regulate the expression of *SlCER6*, a KCS-encoding gene, to synergistically regulate cuticle wax biosynthesis in tomato (Xiong et al., 2020); deposition of wax crystals was observed outside the epidermis after *CsMYB30* was expressed in *Arabidopsis* (Wen et al., 2021). Therefore, we observed the leaf surface morphology of 5-month-old *NtMYB306a*-OE lines with SEM; however, we did not find significant differences in the number and morphology of trichomes between *NtMYB306a*-OE and WT plants. These results indicated that the increased alkanes content in the *NtMYB306a*-OE lines is not due to an increase in trichome density. In addition, we did not observe obvious wax accumulation on the epidermis, and no significant differences was found in cross-section leaves (**Supplementary Figure S9**).

## Conclusion

In this report, we identified NtMYB306a as a member of the S1 subgroup of the R2R3-MYB transcription factor family, and provided evidence to show that NtMYB306a function in wax alkane biosynthesis in trichomes of *N. tabacum*. We showed that NtMYB306a function as activator to regulate the expression of their target genes, including *GDSL, CYP77A2, CYP86A8, ABCG32, KCS1, KCS3, KCS11, KCS20, CER1*, and *CER3*. Besides, the promoter region of NtMYB306a harbored multiple stress-related cis-elements, which positively responded to SA and cold stress during qRT-PCR analysis. Finally, we found that the number and morphology of glandular trichomes were not influenced by NtMYB306a. The *NtMYB306a* can be used as a candidate gene for stress resistance and quality improvement of tobacco.

## Data availability statement

The raw sequencing data generated from this study have been deposited in NCBI Sequence Read Archive (SRA) (http://www.ncbi.nlm.nih.gov/sra) with the accession number PRJNA883934.

## Author Contributions

JY and BL planned and designed research. BW, YG, XZ, MJ and HY performed the experiments. HZ and KK analyzed and discussed the data. JY and DZ wrote the manuscript. JY and KK revised the manuscript. All authors approved the manuscript.

## Funding

This work was supported by the Program of China National Tobacco Corporation Project [110202101005(JY-05)], the Science and Technology project of Guizhou Company of China Tobacco Corporation (2021XM02), the Guizhou Province High-level Innovative Talent Training Program Project ([(2016)4003]), the Guizhou Province Science and Technology Project ([2021]5642), and the National Natural Science Foundation (31860491).

## Conflict of interest

The authors declare that the research was conducted in the absence of any commercial or financial relationships that could be construed as a potential conflict of interest.

## Publisher’s note

All claims expressed in this article are solely those of the authors and do not necessarily represent those of their affiliated organizations, or those of the publisher, the editors and the reviewers. Any product that may be evaluated in this article, or claim that may be made by its manufacturer, is not guaranteed or endorsed by the publisher.
