# Supplementary material for: Cloning, characterization and functional analysis of NtMYB306a gene reveals its role in wax alkane biosynthesis of tobacco trichomes and stress tolerance
Source: Front Plant Sci. 2022 Oct 6;13:1005811. doi: 10.3389/fpls.2022.1005811 (PMC9583951; doi:10.3389/fpls.2022.1005811)
Supplement: Supplementary file 1 [file DataSheet_1.pdf]

Supplementary Material

1 Supplementary Figures

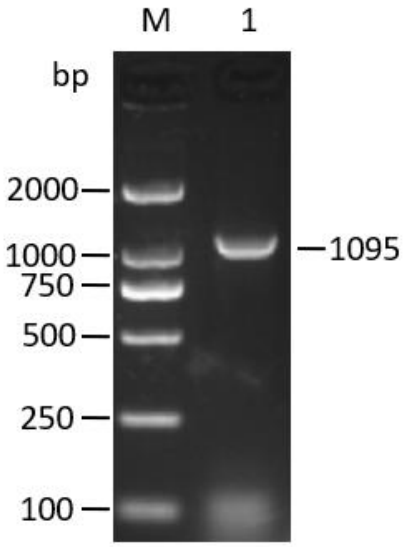

Figure S1. Amplification of full-length *NtMYB306a* CDS. Lane M: DL2000 DNA Marker, Lane 1: Amplification of *NtMYB306a* CDS

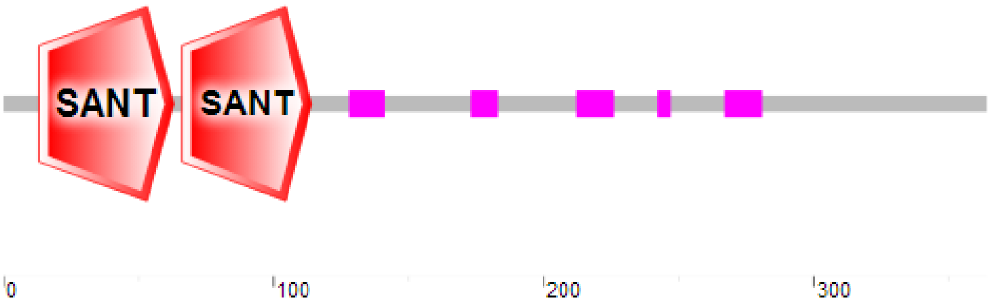

Figure S2. Prediction of conserved domains of NtMYB306a protein. The protein domain of NtMYB306a was predicted by the online website http://smart.embl-heidelberg.de/. SANT represents the two DNA-binding domains R2 (13-63aa) and R3 (66-114aa), respectively. The pink square represents the low complexity, which is 128-141aa, 173-183aa, 212-226aa, 242-247aa, and 267-281aa, respectively.

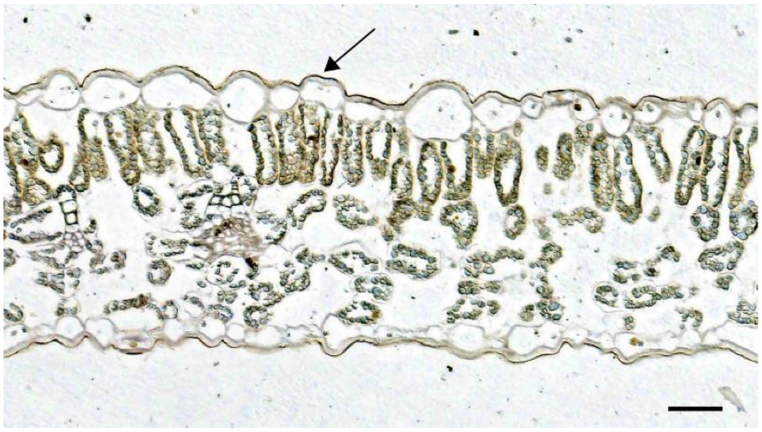

Figure S3. mRNA *in situ* hybridization analysis of *NtMYB306a* in tobacco leaves. Black arrows indicate the epidermal. Scale bars = 50 µm.

GCTCGATGCCTGTCTCATTCGGTCTCGATCGCTCCTGTCCTCGATCTTGACAGGTACCTCGAATCTTGAACTTGGTACGTTATCCTCGTACCTTGGT
CTGATCCACTACGGGGCCGACCTTCGATGCAGCTCCCTGGTCCTGGTCAAATAGAAAAATTGGGTGGGCTCGATTTTAACCGTATACATCATTTTAC
CCTTAATGAAAAATTTTTATAATCACACAAATATTATGGCCCCACAGAGCTTTTACCCATTAAGTTTTTAAGACCATAAATTTTAAAAAAAAAATTT
CTTCTTAAATTTCGTATCGAATCAAACAATCTCATTTAAATTGAAAGGGATGGGGTAACATGTTTCTATCCAACAATGGGGACAAAAGCCAGCTGGT
ACGGTTCACATAAGTTAGGCAATAAAGGTCGTACTCCTCTGAAGTCTCGAGCTATTTATTACAAACTCGAGTACTTACTATGGTTCTTTTCTAGAAA
AATACATTTGAAATTGCGGCCTTTCATCAAATAACAAATACAATACCTATATAATATTTCATTTATCTTATTTTTACTGATAATGTAAGTGAATTTT
TACATGATTAATCTATCATCCCCTACGATATAAATAGTCCATTACTTAAATGGTCACTCACTTATCCGAAATTATTTACTAAAGTCATTTTTCTTTC
GTTTGTAACTAAAAAGTCACTAAACTATATATGCCTATAGCATCAGAAGGTCACTTAACCAGATTTCTCAAATTTTTGATTATCAAATACCAATTTT
ACCCTCTAAATTATCAAGTTTTATCTTCTCCTTTTTTTACTTTTTAAATATTATGATTTTTTCCATTTTAATCTACATTATGGACTTACCTAAGAAA
AAATATATTTTATTTATAAAAAAAAAAAAAAAACTTTTCTAGCTTATATAATATCGAAATAATAATATGATTGAGCATAATTTTAAAGAATATATAAT
GTATATTATAAAAATACCTTTATTTTAAATAATTATTTTTATATTACGTGCATGAAATATATATATTTTACAACTTTTATTTTCTTTCATTCTCAAT
TATGTAAATAAAGTTTTGAATTTTTGTTGTTAATATCAAAATTCTTATGACGGACAAATTGCTCTAATTAATTTTTTTACTATGCTAAATATTTTAT
TATTCTAACATTATATGTGCTTAAATACTATTTTTATTTTTTTAGTTCATAAATAAAATTGGTTTATTCTATAGTAAGTCCATAATATAAATTAAAA
AAAAAACATTATAATATTAAAAAGTTAAAAATAAAAATAAAAAGAAGACAAGAATTGATCATTTAGAGGGTAATATAGGTATTTGGAAATAAACAGT
TCGTAAAATCTAGAGAGTATGACCTTCTAAGTATGACCCTCTATTTTACCTATTTAGAGGTAAAATAGGTATTTTGTTTCAAACAAAAGAAAGATGC
AATTAGTAGATAATTTCGGATAACTAAGTGAGCATTTGAATACTATGGACTCTAGTATAAAAGACCCCATACTTATGTAATAGGAACACGAAATATA
TACACAAACTTAACAAGAACTTTAAGAATGGTAAGTTTAAGAGGATAGTAAAAAAAATTACGTAGGAAAAATGAGAGTAAATTAAATTTATATGTGT
TGCCGGTTGCGTCATAAATGTCGTCAATGCCACATGGTTTAGTGAGGCGGAAAAAGATAAAAAACGATTACTAAAGCATAAGCAAAACAGCTGACTT
TTTAGTGTTTGCAAGCAAATGAGTGTGTTAAATGCTTAATGCTGTCCGCTTTTGACTCATTGAGGATATAGCCCTATTTTACCTCTTCAATCTTTTT
GTCAGCTTAACAACAGCTTTGGTCTCTTCTATCTCCTTCTCTTCTCACTTCTTCAATAATAATATAATAATAATATATATAGACAAGGACCCCCTTT
AGTTAGACGCTTGTCTCAACATTTCTTCATAGGAGTAGTTTCATTATGTCCTATAAAGATATATAGCTAGAGAAAAAAAGCAAGAGAAAGCAGAAAA
CCAATTGTAGTCCTTTTCCCTTTAATTTGTATAGAATAGAATGGGAAGGCCACCT

M G R P P

Figure S4. Sequence of the NtMYB306a promoter (2,077bp). The sequence of the *NtMYB306a* promoter region. The red font indicates the start codon ATG.

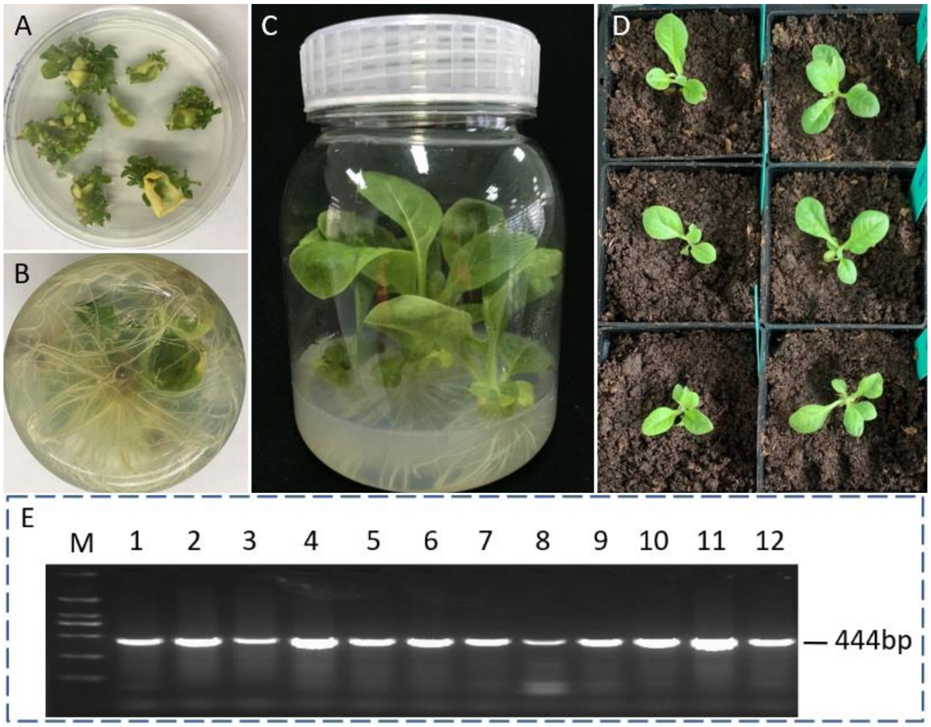

Figure S5. Generation of the *NtMYB306a*-OE tobacco plants and PCR verification of the transformants. (A) Screening and regeneration of resistant plants. **(B-C)** Rooted resistant plants. **(D)** Transplanted plants. **(E)** PCR fragment (444 bp) amplified from transgenic plants. Lane M, DL2000 DNA marker; 1-12 lanes, test samples.

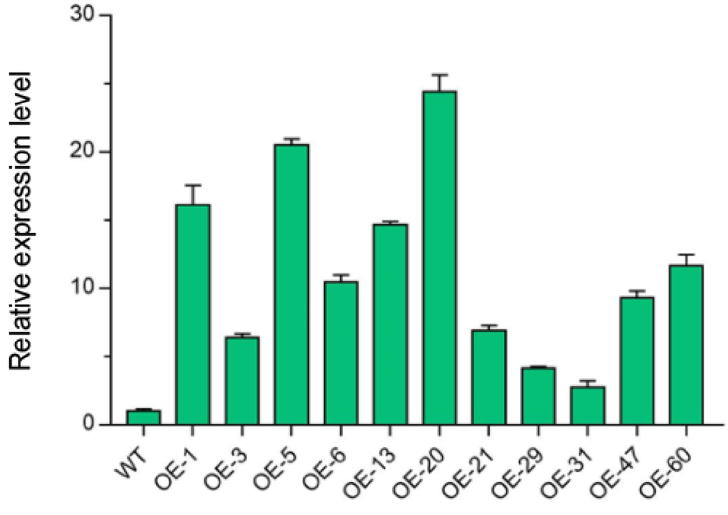

Figure S6. Relative expression levels of *NtMYB306a* in different T0 transgenic lines. Genes were normalized using β-Actin as housekeeping genes. Error bars on each symbol indicate the mean ± SE of three technology replicate reactions.

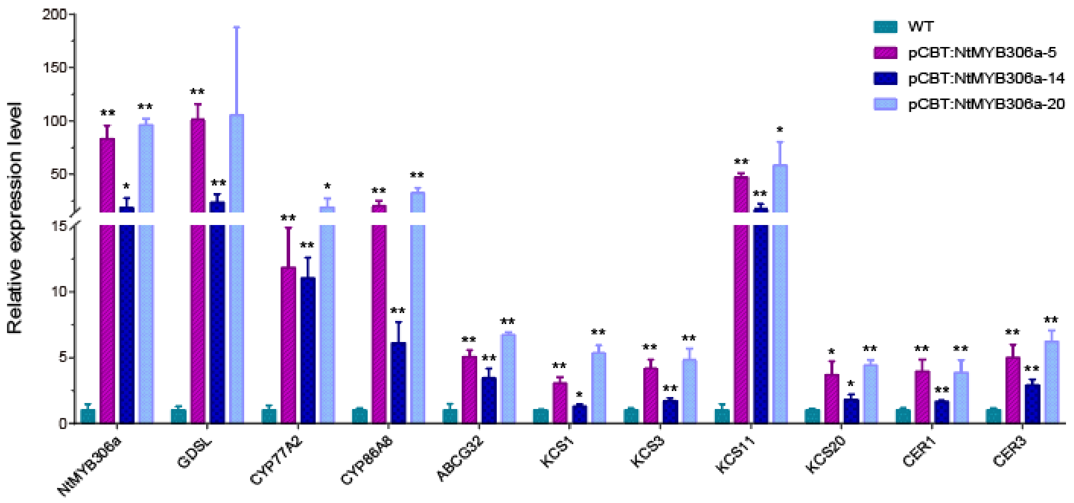

Figure S7. Relative expression levels of ten wax/cutin-related genes in the pCBT:NtMYB306a plants. Error bars indicate ± SD (n = 3). Student's t-test: *, P < 0.05; **, P < 0.01.

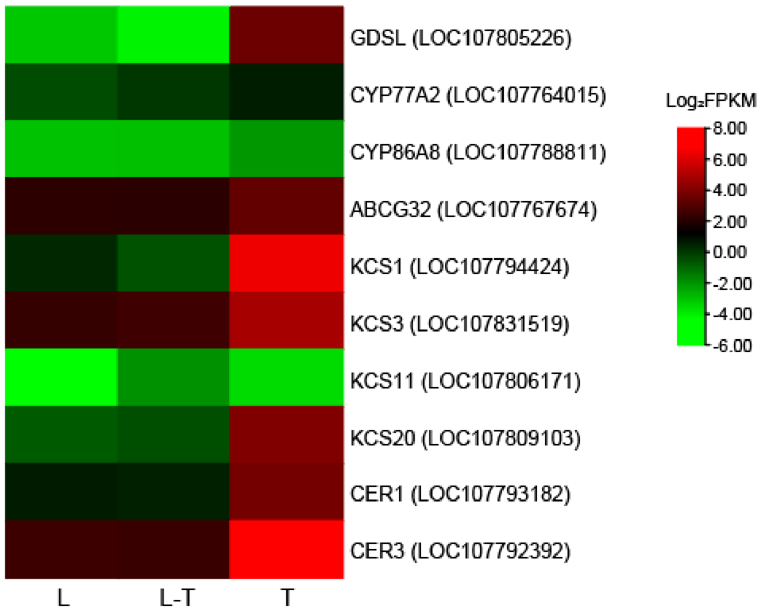

Figure S8. RNA-seq heatmap analysis of ten wax/cutin-related genes in tobacco L, L-T, and T. L: Leaves; L-T: Leaves without trichomes; T: Trichomes**.** Color gradient from low (green) to high (red) represents the value of Log_2FPKM for the selected genes.

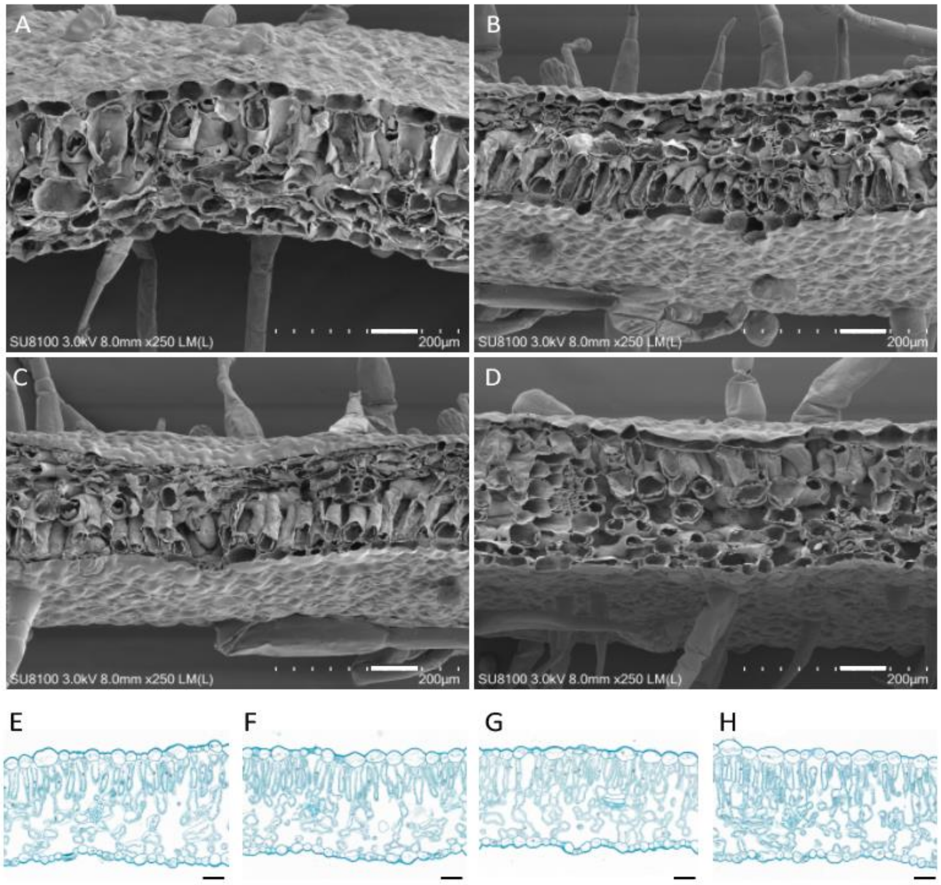

Figure S9. Transection of the leaves in 5-month-old WT and *NtMYB306a*-OE T2 plants. (A-B) Scanning electron micrographs of transection leave, scale bars =50 μm. **(C-D)** Paraffin sections of transection leaves, scale bars = 50 μm. **(A, E)** WT, **(B, F)** OE-3, **(C, G)** OE-13, **(D, H)** OE-20.

2 Supplementary Tables

Table S1. Sequences of the primers used in this study

Primer	Sequence (5'-3')	Purpose
MYB306a-F1	ATGGGAAGGCCACCTTGCTGTGAAA	Gene clone
MYB306a-R1	TCAAAACAAATCTGCAGTTTCACTT	Gene clone
pNtMYB306a-GUS-F1	ggatccGCTCGATGCCTGTCTCATTCGGTCT	Promoter clone and GUS analysis
pNtMYB306a-GUS-R1	ccatggTCTATTCTATACAAATTAAAGGGAAAAG	Promoter clone and GUS analysis
Actin-Q-F	CGGAATCCACGAGACTACATAC	qRT-PCR
Actin-Q-R	GGGAAGCCAAGATAGAGC	qRT-PCR
MYB306-Q-F1	TCTAGCCCTAGCGAAGGAACAA	qRT-PCR
MYB306-Q-R1	CACCCTCATCGACCGACATG	qRT-PCR
GDSL-Q-F2	AATAGGAGAGCCAGCGACAATAA	qRT-PCR
GDSL-Q-R2	TGGAGAGAACAGGCGTGAGATAA	qRT-PCR
CYP77A2-Q-F1	CGAAAAGGACATAGAGAAAATGCC	qRT-PCR
CYP77A2-Q-R1	TCAGCGAAAAGTACGTAGGAGGAT	qRT-PCR
CYP86A8-Q-F1	GGTTCACCTTCATCTCAAGATCTCT	qRT-PCR
CYP86A8-Q-R1	TGTCTGGTACGTGCCACCACAC	qRT-PCR
ABCG32-Q-F1	GGGGGTTTTGGATTTCTCCC	qRT-PCR
ABCG32-Q-R1	GCTCTCTTGTCCCATATGTGCC	qRT-PCR
KCS1-Q-F1	TCCAACCCCATCTCTCTCCTC	qRT-PCR
KCS1-Q-R1	CACTACAGCCCATGCCACCT	qRT-PCR
KCS3-Q-F1	CTCCCAGTGAGAGAGTTACTTCGAT	qRT-PCR
KCS3-Q-R1	GCCTAGCTCCTCCTTTTGTTGA	qRT-PCR

KCS11-Q-F1	TGCATGGATTTAGCAAGAAAAGAGG	qRT-PCR
KCS11-Q-R1	CGGCACAGGATGAAAAACAGAG	qRT-PCR
KCS20-Q-F1	TAATGGCTGTAGCAGGAGAGGC	qRT-PCR
KCS20-Q-R1	AGTAGTTGTTCGGACATAGGAAGGA	qRT-PCR
CER1-Q-F1	TCTGGTGTTAGATTGGTTGAGGC	qRT-PCR
CER1-Q-R1	GAAAAGGGGAGAAAATTGAGGACTT	qRT-PCR
CER3-Q-F1	CCTCCTGTCATTCCCTTTCGC	qRT-PCR
CER3-Q-R1	CTACGCCTCTTCCCATTGTATACTC	qRT-PCR
MYB306a-SL-F1	cagtGGTCTCacaacatgggaaggccaccttgctg	Subcellular localization
MYB306a-SL-R1	cagtGGTCTCatacaaaacaaatctgcagtttcac	Subcellular localization
MYB306a-Y2H-F1	ggacctgcatatggcATGGGAAGGCCACCTTGCT	Transactivation assay
MYB306a-Y2H-R1	tatgcggccgctgcaTCAAAACAAATCTGCAGTTTCACT	Transactivation assay

Table S2. Putative *cis*-acting regulatory elements identified in the promoter sequence of *NtMYB306a* using the PlantCARE database

No.	Cis-acting elements	Number	Characteristic sequence	Biological function
1	ABRE	1	ACGTG	cis-acting element involved in the abscisic acid responsiveness
2	ARE	3	AAACCA	cis-acting regulatory element essential for the anaerobic induction
3	Box 4	2	ATTAAT	part of a conserved DNA module involved in light responsiveness
4	CAAT-box	35	CAAT/CAAAT/CCAAT/CCCAATTT	common cis-acting element in promoter and enhancer regions
5	CGTCA-motif	3	CGTCA	cis-acting regulatory element involved in the MeJA-responsiveness
6	G-box	1	TACGTG	cis-acting regulatory element involved in light responsiveness
7	GCN4_motif	1	TGAGTCA	cis-regulatory element involved in endosperm expression
8	GT1_motirf	2	GGTTAA	light responsive element
9	I_box	1	TGATAATGT	part of a light responsive element
10	LTR	2	CCGAAA	cis-acting element involved in low-temperature responsiveness
11	MBSI	1	aaaAaaC(G/C)GTTA	MYB binding site involved in flavonoid biosynthetic genes regulation
12	MYB	4	TAACCA/CAACAG	MYB-binding site
13	MYC	5	CATTTG/CAATTG/CATGTG	MYC-binding site
14	TATA-box	79	ATATAA/ATATAT/ATTATA/ccTATAAaa/ TATA/TATAA/TATAAA/TATAAAA/ TATAAAT/TATAAATA/TATACA/TATATA/ TATATAA/TATTTAAA	core promoter element around -30 of transcription start
15	TCA-element	1	CCATCTTTTT	cis-acting element involved in salicylic acid responsiveness
16	TGACG-motif	3	TGACG	cis-acting regulatory element involved in the MeJA-responsiveness
17	chs-CMA1a	1	TTACTTAA	part of a light responsive element
